# Microstructural Evolution and Surface Mechanical Properties of the Titanium Alloy Ti-13Nb-13Zr Subjected to Laser Shock Processing

**DOI:** 10.3390/ma16010238

**Published:** 2022-12-27

**Authors:** Jiajun Wu, Xingze Lin, Hongchao Qiao, Jibin Zhao, Wangwang Ding, Ran Zhu

**Affiliations:** 1 College of Engineering, Shantou University, Shantou 515063, China; 2 State Key Laboratory of Robotics, Shenyang Institute of Automation, Chinese Academy of Sciences, Shenyang 110016, China; 3 Institute for Advanced Materials and Technology, University of Science & Technology Beijing, Beijing 100083, China; 4 College of Mechanical and Electronic Engineering, Shandong University of Science and Technology, Qingdao 266590, China

**Keywords:** laser shock processing, titanium alloy Ti-13Nb-13Zr, surface topography, mechanical properties, microstructure

## Abstract

As a progressive surface-hardening technology, laser shock processing (LSP) can enhance the mechanical properties and extend fatigue life for metallic components through laser-generated high-pressure plasma shock waves. In this work, LSP was used to treat titanium alloy Ti-13Nb-13Zr experimental coupons, and the microstructural response and surface mechanical properties of the Ti-13Nb-13Zr experimental coupons were investigated. After the LSP treatment, the X-ray diffraction (XRD) peaks were shifted without any new phase formation. The surface roughness of the experimental coupons increased, which can be explained by the LSP-induced severe plastic deformation. The LSP treatment effectively enhanced the surface compressive residual stress of Ti-13Nb-13Zr. Meanwhile, the microhardness of the Ti-13Nb-13Zr was also obviously increased after the LSP treatment. The experimental results also showed that the number of shocks times is an important factor in the improvement of surface mechanical properties. LSP treatment with multiple shocks can lead to more severe plastic deformation. The surface roughness, surface compressive residual stress and microhardness of the Ti-13Nb-13Zr experimental coupons shocked three times are higher than those after one shock. What is more, grain refinement accounts for the mechanical properties’ enhancements after the LSP treatment.

## 1. Introduction

Given its excellent biocompatibility, corrosion resistance, lower elastic modulus and outstanding mechanical performances, titanium alloy Ti-13Nb-13Zr has been widely applied in clinical applications [1]. At present, common biomedical titanium alloy parts mainly consist of total hip, artificial spines, embracing plates, and dental implants, etc. However, these parts might fail due to different causes such as cyclic load conditions [2], fracture [3], aseptic loosening [4], wear and corrosion [5]. These failures always require high-risk and high-cost replacement surgery. With the purpose of avoiding or delaying the need for a second surgery, the fatigue life of titanium alloy components needs to be enhanced. According to existing literature reports, mechanical surface-hardening technologies can be regarded as a useful method of increasing the metallic materials’ mechanical properties and extending the components’ fatigue life [6]. The common surface-hardening technologies mainly include shot peening [7], ultrasonic peening [8], rolling [9], water-jet peening [10] and ultrasonic nanocrystal surface modification [11]. The important feature of these technologies is that they introduce beneficial compressive residual stress in near-surface layers without changing the chemical reactions and structures [12]. However, the effective depth of beneficial compressive residual stress introduced by these techniques is about 300 μm at most [13], which is unsuitable for high-performance materials and advanced equipment.

Laser shock processing (LSP) is a progressive metal surface-hardening technology, which can increase the mechanical properties, wear resistance, fatigue performance and corrosion resistance of metallic components through high-pressure laser-generated plasma shock waves [14,15,16]. Prior to LSP treatment, the surface to be shocked usually needs to be ground and polished with the purpose of obtaining a nearly non-scratch surface. In order to protect the shocked surface from laser thermal ablation, the surface needs to be covered with an absorbing protective layer. In addition, with the aim of maintaining or improving the peak pressure for plasma shock waves, the material’s surface is also required to be covered by a vitreous constraint layer. During LSP, the high-energy nanosecond pulse energy transmits through the vitreous constraint layer and acts on the material’s surface; the covered absorbing protective layer absorbs the laser energy, causing the target material temperature to rise. At the same time, laser-induced plasma is formed [17]. The plasma continues to absorb the laser energy and expands quickly, which will lead to the formation of laser-induced plasma shock waves with a pressure level of GPa [18]. The peak pressure of laser-induced plasma shock waves is greater than the dynamic yield strength of the targeted material. As a result, severe plastic deformation (SPD) will take place in the material’s near-surface [19]. It is well known that the SPD can refine material grains and induce beneficial compressive residual stress in the near-surface layer [20]. Thus, the mechanical properties of the material will be improved and the fatigue performance of components will be extended significantly [21]. Compared to the common surface-hardening technologies, LSP has more advantages, such as an excellent hardening effect, greater working efficiency and better structure adaptability [22]. Therefore, applying LSP technology to treat the titanium alloy Ti-13Nb-13Zr offers significant benefits for the enhancement of mechanical properties and fatigue performance.

In recent years, LSP treatment has been gradually applied across the fields of biomedical engineering. For instance, Shen et al. [23] studied the effects of laser pulse energy and overlap rates on surface topography, residual stress and welting performance of biomedical titanium alloy Ti-6A1-7Nb. The experimental results showed that the surface roughness and introduced compressive residual stress were founded to be raised with laser pulse energy and overlap rate, but the changes in relation to contact angle and surface free energy were just the opposite. Huang et al. [24] investigated the effect of LSP treatment on friction and wear performance of titanium alloy Ti-13Nb-13Zr. They found that the mass loss and friction coefficient of the experimental coupons were reduced significantly after LSP treatment. According to the findings from Guo et al. [25], the corrosion resistance of biodegradable metallic implants was improved significantly by LSP. These previous studies mainly focused on the improvement in mechanical performance of biomedical titanium alloy subjected to LSP. In fact, these improvements are often determined by the experimental parameters of LSP treatment and related to microstructural evolution. Of course, there is little literature reporting investigations of the microstructural evolution and mechanical performance of Ti-13Nb-13Zr subjected to LSP.

In this work, the SIA-LSP-23 series LSP system was used to treat titanium alloy Ti-13Nb-13Zr experimental coupons: laser energy of 6J, an overlap rate of 50%, pulsed width of 15ns and spot diameter of 3 mm were used with one and three shocks. The microstructural response and surface mechanical properties of the Ti-13Nb-13Zr experimental coupons were investigated. This work has important reference value and guiding significance for researchers to apply LSP treatment to modify the surface features and enhance the mechanical performance of Ti-13Nb-13Zr.

## 2. Experimental Procedures

### 2.1. Material Preparation

The titanium alloy Ti-13Nb-13Zr experimental material used in this work was commercially bought from Baoji Intercity Titanium Nickel Co., Ltd. (Intercity Titanium Nickel Co., Ltd., Baoji, China) in form of a plate with thickness of about 10 mm. The nominal composition of titanium alloy Ti-13Nb-13Zr is listed in Table 1 [26]. The experimental coupons were cut into a square shape with the dimensions of 30 mm × 30 mm × 5 mm (length, width and thickness) by electronic discharge machining (EDM). Before the LSP experiment, the surface to be shocked of these experimental coupons were ground from 60 grid to 1200 grid using silicon carbide abrasive papers, and then mechanically polished.

### 2.2. LSP Experiment Setup

**Figure 1:**
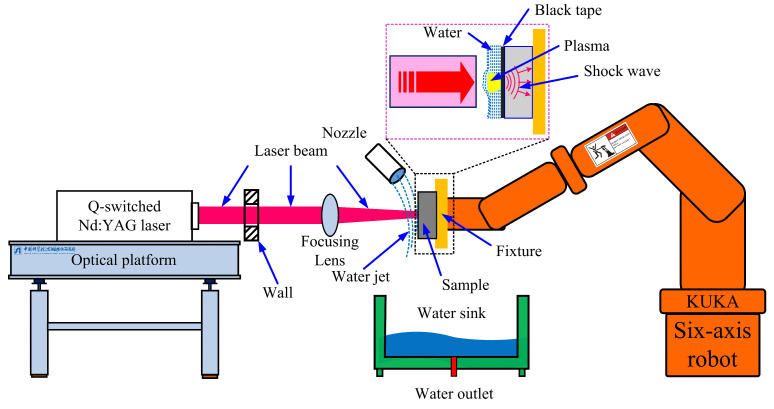
The schematic diagram of LSP treatment employed in this work.

The LSP experiment was performed with the SIA-LSP-23 series LSP system, developed by Shenyang Institute of Automation, Chinese Academy of Sciences (Shenyang, Liaoning, China). A Q-switched Nd:YAG laser is used to provide the laser source, and its main parameters are shown in Table 2. The output circular laser beam with an initial diameter of 32 mm travelled through wall and focusing lens in succession, and then irradiated the coupon’s surface with a circular laser beam spot. The spot diameter of the laser beam acting on the surface can be controlled by adjusting the distance between the Nd:YAG laser and shocked surface of the coupon [27]. In this work, a laser energy of 6 J, overlap rate of 50%, pulsed width of 15 ns and spot diameter of 3 mm were used with one and three shocks. The 130 μm thick black tape from Nitto Denko Inc. (Osaki, Tokyo, Japan) was used as the absorbing protective layer, while the running deionized water was selected as the absorbing constraint layer. Figure 1 shows the schematic diagram of LSP employed in this work.

The laser power density can be calculated as follows [28].

(1) $$I=\frac{4E}{\pi d^{2}\tau }$$

where *I* represents laser power density, *E*, *τ* and *d* represent laser energy, pulse width and spot diameter for the laser beam acting on the shocked surface of the coupon, respectively. Thus, the laser power density applied in this work is 5.66 GW/cm^2^.

### 2.3. Characterization Methods

The surface topography of the experimental coupons before and after the LSP treatment was measured by the Contour GT-K 3-D optical microscopes system (Bruker Nano Inc., Tucson, AZ, USA). The surface topography mapping was carried out with ×5 lens and ×7 magnification. The scanning strategy applied in this work was to start from the left corner on the surface of experimental coupons, and then use a zigzag route to obtain a rectangular map with the size of 12 mm × 12 mm at the end.

X-ray diffraction (XRD) was carried out to study the phase structure and crystal orientation of experimental coupons prior and after the LSP treatment by using an X’PERT POWDER X-ray diffractometer (Malvern Panalytical Inc., Almelo, The Netherlands) with radiation source of Cu_Kα. The scanning 2θ angle ranged from 10° to 90°, while the scanning rate of 4.0°/min was selected.

The Harke-Spca-X2 contact angle measuring instrument (HARKE, Beijing, China) was selected to determine the contact angle of the experimental coupons before and after the LSP treatment with a 1.5 μL distilled water droplet at room temperature. The sessile drop method was selected for this work.

A PROTO LXRD X-ray residual stress measuring instrument (PROTO Mfg. Ltd., Oldcastle, ON, Canada) was used to the measure surface residual stress of the experimental coupons. The X-ray residual stress measurement parameters applied in this work are listed in Table 3.

The experimental coupons’ microhardness before and after the LSP treatment was determined by the FM-310 Vickers microhardness tester (Future-tech Enterprise Inc., Nagashima, Japan); an indentation load of 1.98 N and dwelling time of 15 s were selected for this work. In-depth microhardness was determined from the top of the surface to the depth of 1mm on the cross-section surface.

The EBSD analysis was carried out on the cross-section surface of the experimental coupons, performed using a SEM instrument (TESCAN MIRA3, Brno, Czech) with an HKL-EBSD operating system at the working voltage of 25 kV. The measuring step size for the orientation imaging microscopy in this work was 0.6 μm.

## 3. Experimental Results and Discussion

### 3.1. Surface Topography Evolution and Contact Angle

**Figure 2:**
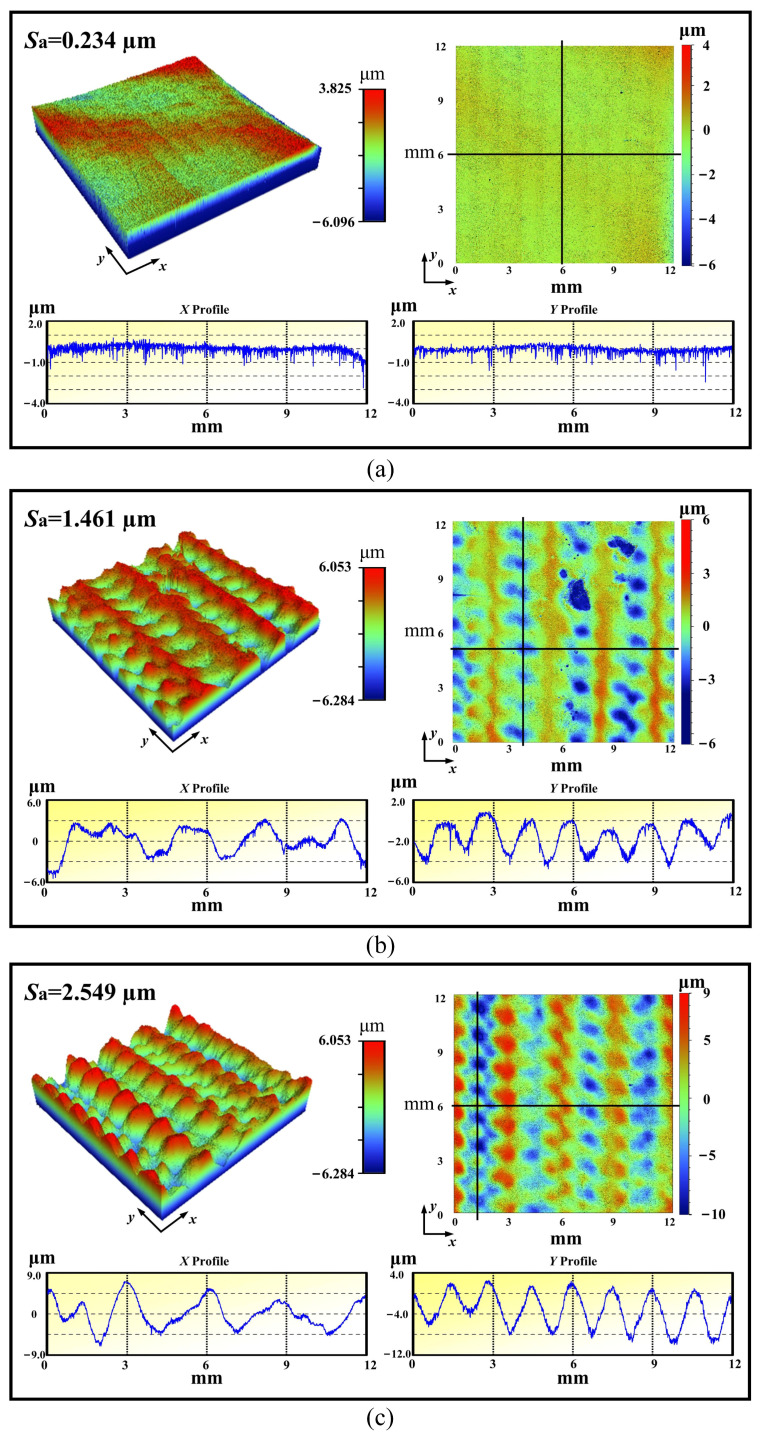
Surface topographies for Ti-13Nb-13Zr experimental coupons: (**a**) untreated; (**b**) 6J-1time; and (**c**) 6J-3times.

Since SPD is induced by LSP in the near-surface, this will cause surface topography evolution. Figure 2 displays the surface topographies for Ti-13Nb-13Zr experimental coupons before and after LSP treatment, and consists of the 3-D and 2-D surface topography, and the cross-sectional profile in x direction and y direction. In this work, the mean 3D surface roughness *S*_a_ was selected to evaluate the surface roughness, which can be expressed as follows.

(2) $$S_{a}=\frac{1}{A}\iint_{A}\left|Z\left(x,y\right)\right|dxdy$$

where *A* is the area of the measuring region, and *Z*(*x*, *y*) is the height of data point (*x*, *y*) relative to the referenced plane.

Before LSP treatment, the shocked surface of the experimental coupon was treated by a mechanical grind and polish process, so the surface roughness is relatively low. From Figure 2 it can be seen that the surface roughness of the untreated experimental coupon was 0.234 μm. After the LSP treatment, the surface roughness increased sharply. When shocked once, the LSP condition of 6J-1time, the surface roughness of the experimental coupon increased to 1.461 μm. When shocked three times, the LSP condition of 6J-3times, the surface roughness of the experimental coupon is increased to 2.549 μm. Using the same laser power density, multiple shocks results in more serious plastic deformation induced by LSP, resulting in the increase of surface roughness. Surface topographies, such as grooves and dimples in the experimental coupons after LSP treatment, are quite similar. However, since the number of shocks is different, this will lead to a difference of surface morphology, especially at the Z-profile.

**Figure 3:**
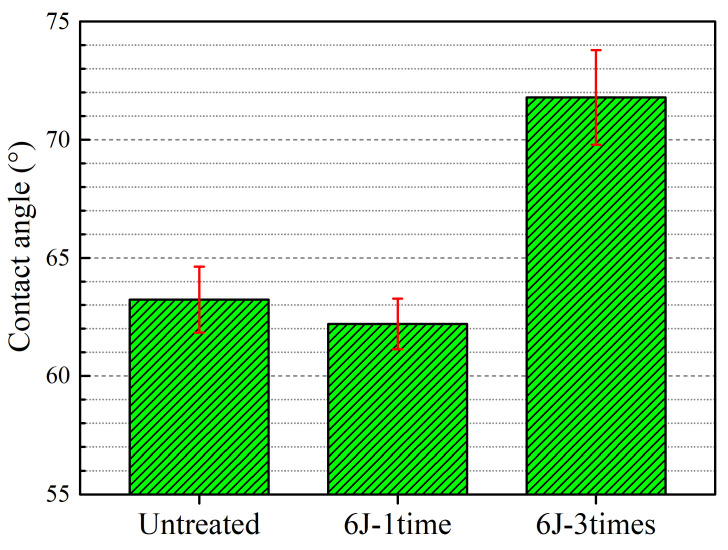
Contact angle of experimental coupons.

A specific surface at a designated temperature and pressure has a specific equilibrium contact angle [16]. The wettability is closely related to the contact angle. In clinical applications, the surface wettability of metallic manufactured implants is a very important parameter, which needs to be measured. The contact angle of the experimental coupons is shown in Figure 3. As shown in the Figure, the average contact angle for an untreated experimental coupon is about 63.2°, whereas that for LSP-treated experimental coupons is about 62.2° for 6J-1time, and 71.8° for 6J-3times. According to the previous reports [16,23,28], the contact angle of a metallic material treated by LSP is proportional to the surface roughness. However, the average contact angle of a 6J-1time experimental coupon is lower than that of an untreated experimental coupon, but that of a 6J-3times experimental coupon is much higher than that of an untreated experimental coupon. Thus, the law of contact angles is shown to be associated with the properties of a material, and the difference noted above and related mechanism need to be investigated in detail in a future work. Overall, it can be inferred that LSP can create a high surface roughness, and increases in the shock times can lead to a highly hydrophobic surface on the Ti-13Nb-13Zr alloy without any damage or metal oxide formation.

### 3.2. XRD Analysis

**Figure 4:**
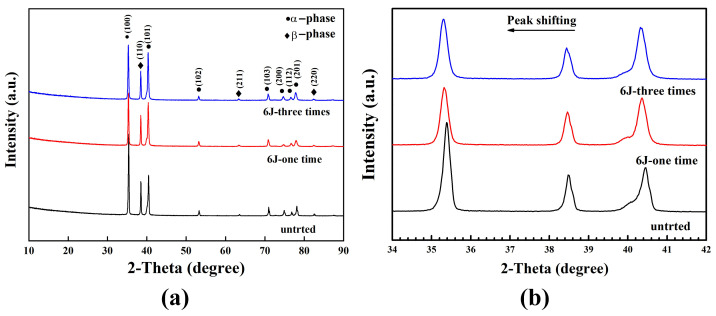
(**a**) XRD patterns of Ti-13Nb-13Zr coupon surfaces before and after the LSP treatment with 6J-1time and 6J-3times; (**b**) diffraction peak shifting for α(100), β(110) and α(110) planes.

The XRD patterns on the coupon surface of untreated and LSP-treated titanium alloy Ti-13Nb-13Zr after 6J-1time and 6J-3times is presented in Figure 4. Bragg diffraction peaks of *α*(100), (101), (102), (103), (200), (112), (201) and *β*(110), (211), (220) were indexed in Figure 4a. It is worth noting that the contents for *α* phase are greater than that for β phase in coupons of Ti-13Nb-13Zr. What is more, there is no new phase generation after LSP treatment. But the diffraction peaks such as the *α*(100), *β*(110) and *α*(110) planes are shifted to a smaller angle (see Figure 4b for detail), which can be explained by the residual stress introduced by the LSP effect [18,29]. It can be inferred that LSP treatment cannot lead to the formation of oxide or damage or the alteration of crystal structure for Ti-13Nb-13Zr coupons.

### 3.3. Surface Residual Stress

**Figure 5:**
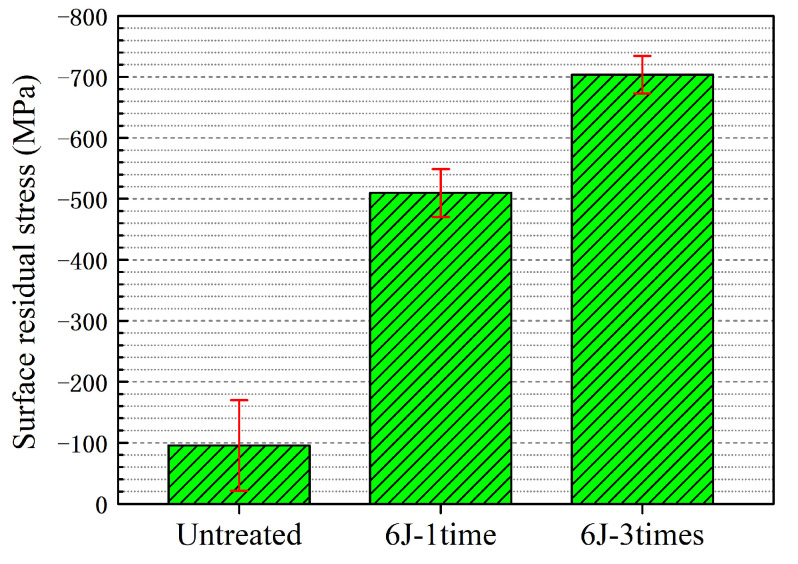
Surface residual stress of Ti-13Nb-13Zr coupon before and after the LSP treatment with 6J-1time and 6J-3times.

Residual stress is one of the important mechanical property parameters for materials, existing in almost all manufactured components or structures [30]. One of the advantages of LSP is the introduction of a stable beneficial compressive residual stress layer in the materials’ near-surface through laser-induced plasma shock waves with a pressure level of GPa. Enhancement of the fatigue performance with LSP treatment can be positively affected by compressive residual stress. Hence, analysis of the residual stress of the experimental coupons before and after LSP treatment is a necessary and important step. Figure 5 depicts the surface residual stress of Ti-13Nb-13Zr coupons before and after LSP treatment with 6J-1time and 6J-3times. A compressive residual stress with value of −95.5 MPa existed on the surface of untreated experimental coupons. The compressive residual stress present in the untreated experimental coupon was mainly caused by grinding and polishing. A similar observation can be seen in previous studies [16,31]. A higher level of compressive residual stress can be observed after the LSP treatment. The value of surface residual stress for 6J-1time experimental coupons and 6J-3times experimental coupons were −509.8 MPa and −703.9 MPa, respectively. The introduction of beneficial compressive residual stress of coupons subjected to LSP can be attributed to the interplanar spacing [18] and the SPD accompanying the microstructural evolution under laser-induced shock waves with a pressure level of GPa [16,23]. Multiple shocks can remarkably enhance the surface compressive residual stress of the coupons. 

### 3.4. Microhardness

**Figure 6:**
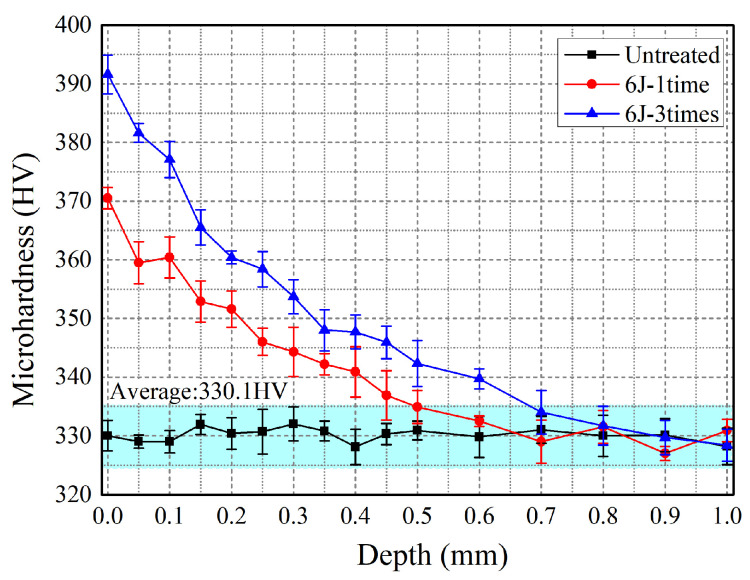
In-depth microhardness distribution of experimental coupons before and after the LSP treatment for 6J-1time and 6J-3times.

Microhardness is also an important property parameter for materials, usually applied to investigate the elastic/plastic deformation mechanism [30]. Figure 6 illustrates the in-depth microhardness distribution of Ti-13Nb-13Zr coupons before and after the LSP treatment for 6J-1time and 6J-3times. As for the untreated experimental coupon, the measured in-depth microhardness curve was almost a straight line except for small wiggles, and the average value is 330.1 HV. After the LSP treatment, the microhardness of the Ti-13Nb-13Zr coupon is increased. For the 6J-1time experimental coupon, the surface microhardness can be reached at a maximum value of 370.5 HV and then gradually decreases to about 330 HV when the depth is increased to about 0.65 mm. For the 6J-3times experimental coupon, the surface microhardness can be reached at a maximum value of 391.6 HV and then gradually decreases to about 330 HV when the depth is increased to about 0.87 mm. From Figure 4, it can be observed that the surface microhardness of the Ti-13Nb-13Zr coupon is increased by 12.23% after the LSP treatment for one shock, whereas it increases by 5.70% from one shock to three shocks. In addition, LSP treatment generates several hundred micrometers of working hardened layer in the near-surface layer for the Ti-13Nb-13Zr coupon. The depth of the working hardened layer increased from about 0.65 mm to 0.87 mm when the number of shocks was varied from one to three. Therefore, select multiple shock times can enhance the values for surface microhardness and the corresponding depth for the working hardened layer remarkably. In addition, LSP treatment with multiple numbers of shocks can be regarded as a quite practicable means to achieve greater microhardness and a deeper working hardened layer [32].

### 3.5. Microstructural Evolution

**Figure 7:**
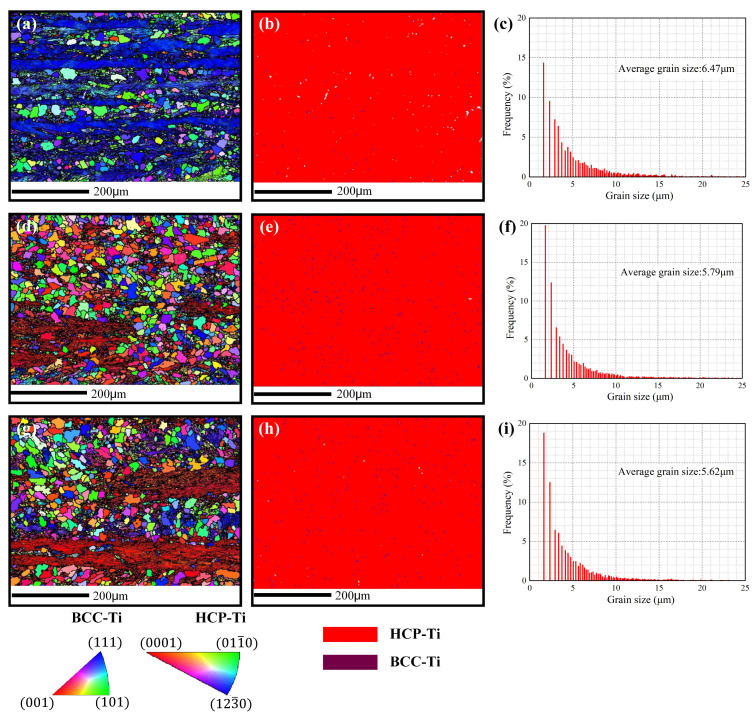
Cross-sectional IPF images, phase distribution and grain size distribution of experimental coupons before and after LSP: (**a**–**c**) experimental coupon before LSP treatment; (**d**–**f**) LSP experimental coupon after LSP treatment with one shock; (**g**–**i**) LSP experimental coupon after LSP treatment with three shocks.

**Figure 8:**
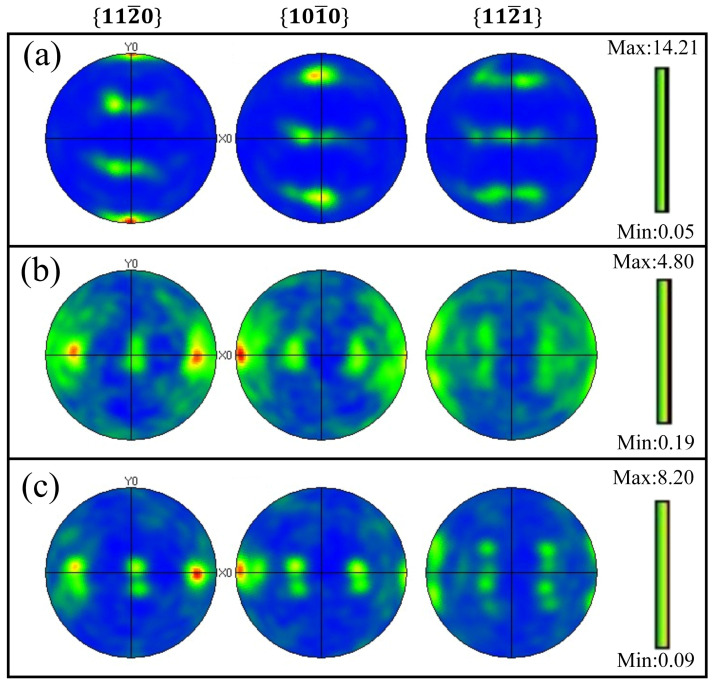
EBSD pole figures of experimental coupons before and after LSP treatment: (**a**) untreated, (**b**) 6J-1time, and (**c**) 6J-3times.

The SPD behavior induced by LSP can be expressed by the microstructural features, which were formed as the laser-induced plasma shock wave propagated to the matrix surface. The cross-sectional IPF images, phase distribution and grain size distribution of experimental coupons before and after LSP is shown in Figure 7, which captures the evolution of microstructure. The experimental coupon before LSP treatment displays near-equiaxial grain, while its average grain size is about 6.47 μm. After the LSP treatment, the grain refinement area appeared on the matrix near the treated surface (Figure 7d,g), while refined area increased with the numbers of shocks. Meanwhile, the average grain size of LSP experimental coupons with one and three shocksdecreased to 5.79 μm and 5.62 μm, respectively. In addition, all experimental coupons mainly consist of HCP phase and a few of BCC phase (Figure 7b,e,h). This reveals that LSP treatment cannot change the phase content, which correspondsto the XRD results in Figure 4. Further, the pole figures are applied to experimental coupons before and after the LSP treatment for 6J-1time and 6J-3times to measure textural evolution, which is displayed in Figure 8. Compared to the experimental coupon before the LSP treatment, a weaker preferred orientation occurred in the experimental coupons after the LSP treatment. The situation may arise because of the grain refinement and change of the grain orientation (Figure 7 and Figure 8).

It is well known that the enhancement of the microhardness is mainly related to the increase of the dislocation density [32,33]. Therefore, the microhardness can be expressed by the microstructure, that is dislocation density. Microhardness *H* can be expressed by Equation (3).

(3) $$H=H_{0}+\alpha Gbp$$

where *H*_0_ is the initial microhardness for the material, *α*, *G*, *b* and *p* represent the material’s constants, the shear modulus, the Burgers vector value and the dislocation density, respectively. For a determined material, the value of *α*, *G* and *b* are determined. Thus, the microhardness is proportional to the dislocation density. 

During the LSP process, grain refinement is considered as the preferred plastic deformation modulus, and the dislocation density can be determined by Equation (4).

(4) p=Δθ/(db)

where *∆θ* represents the deviation angle, which is induced by excess dislocations, and d represents the M-T spacing. As observed from the results in Figure 7, it can be shown that the value of d is decreased after LSP, and with stronger LSP conditions, its value is smaller. Hence, the LSP treatment can cause grain refinement, which will lead to enhancement of the microhardness.

## 4. Conclusions

In this work, LSP treatment was used to treat titanium alloy Ti-13Nb-13Zr experimental coupons, and the microstructural response and surface mechanical properties of the Ti-13Nb-13Zr experimental coupons were investigated. The main findings are as below:

(1) After the LSP treatment, the XRD diffraction peaks were shifted without any new phase formation. 

(2) The SPD induced by LSP can lead to surface topography evolution and enhance the surface roughness, and multiple shock times can lead to a high surface roughness and a highly hydrophobic surface.

(3) LSP treatment can lead to improvement of the surface compressive residual stress and microhardness. Surface compressive residual stress for untreated experimental coupons is −95.5 MPa, while surface residual stress for 6J-1time and 6J-3times experimental coupons are −509.8 MPa and −703.9 MPa. Surface microhardness is increased by 12.23% after the LSP treatment with one shock, whereas it is increased by 5.70% as the number of shocks is raised from one to three. 

(4) Grain refinement appeared on the matrix near the LSP treated surface, while grain size decreased from 6.47 μm (before LSP treatment) to 5.79 μm (6J-1time) and 5.62 μm (6J-3times), respectively. Meanwhile, LSP treatment led to a weaker preferred orientation of Ti-13Nb-13Zr. In addition, grain refinement accounts for the mechanical properties’ enhancements after the LSP treatment.

## Figures and Tables

**Table 1:** Nominal composition of titanium alloy Ti-13Nb-13Zr [26].

Element	Ti	Nb	Zr	C	Fe	N	O	H	S
**Wt%**	Bal.	13.18	13.49	0.035	0.085	0.019	0.078	0.055	<0.001

**Table 2:** Technical parameters for Q-switched Nd:YAG laser.

Technical Parameters	Values
Working medium	Nd:YAG
Wavelength	1.064 μm
Laser energy	2–20 J
Working frequency	0–5 Hz
Pulse width (FWHM)	10 ns–15 ns
Shape of laser beam spot	Circle (roundness >95%)
Initial output spot diameter	32 mm
Laser beam profile	Three dimensional flat-top distribution
ASE energy	<20 mJ

**Table 3:** X-ray stress measurement parameters applied in this work.

Measurement Parameters	Value
X-ray tube	Cu_K-Alpha
Diffraction plane	hkl-311
Working voltage	25 KV
Electric current	20 mA
Oscillation angle	±5°
Wavelength	1.5418380 Å
Exposure duration	1 s
Exposure times	10 s
Peak position determination method	Half-maximum intensity
Irradiation spot diameter	2 mm

## Data Availability

All data supporting the conclusions of this manuscript are included within the manuscript.
